# Genetic Differentiation and Relationship among *Castanopsis chinensis*, *C. qiongbeiensis*, and *C. glabrifolia* (Fagaceae) as Revealed by Nuclear SSR Markers

**DOI:** 10.3390/plants13111486

**Published:** 2024-05-28

**Authors:** Yang Wu, Kai Yang, Xiangying Wen, Ye Sun

**Affiliations:** 1 Guangdong Key Laboratory for Innovative Development and Utilization of Forest Plant Germplasm, College of Forestry and Landscape Architecture, South China Agricultural University, Guangzhou 510642, China; yangwuhcy@163.com (Y.W.); jerrykai1314@163.com (K.Y.); 2 South China Botanical Garden, Chinese Academy of Sciences, Guangzhou 510650, China

**Keywords:** *Castanopsis*, microsatellite marker, geographical isolation, gene flow

## Abstract

*Castanopsis chinensis* (Spreng.) Hance is widespread in the subtropical forests of China. *Castanopsis qiongbeiensis* G.A. Fu and *Castanopsis glabrifolia* J. Q. Li & Li Chen are limited to the coastal beaches of Wenchang county in the northeast of Hainan Island, and have similar morphological characteristics to *C. chinensis*. It is supposed that *C. qiongbeiensis* and *C. glabrifolia* are closely related to *C. chinensis*. In the present study, the genetic differentiation, gene flow, and genetic relationship of *C. chinensis*, *C. qiongbeiensis*, and *C. glabrifolia* were investigated by using 15 nuclear microsatellite markers; a total of 308 individuals from 17 populations were sampled in the three species. The allelic variation of nuclear microsatellites revealed moderate but significant genetic differentiation (F_CT_ = 0.076) among *C. chinensis*, *C. qiongbeiensis*, and *C. glabrifolia*, and genetic differentiation between *C. chinensis and C. glabrifolia* was larger than that between *C. chinensis* and *C. qiongbeiensis*. Demographic simulations revealed unidirectional gene flow from *C. chinensis* to *C. glabrifolia* and *C. qiongbeiensis*, which highlight dispersal from mainland to island. The isolation effect of Qiongzhou Strait increased the genetic differentiation of species on both sides of the strait; however, the differentiation was diminished by gene flow that occurred during the historical period when Hainan Island was connected to mainland China. Our results supported the argument that *C. glabrifolia* should be considered an independent species and argued that *C. qiongbeiensis* should be regarded as an incipient species and independent conservation unit.

## 1. Introduction

Genetic diversity and population structure are important components of biodiversity conservation, which underlie the evolutionary potential of species and are crucial for the survival and environmental adaptability of populations [1]. Understanding genetic structure and population differentiation has been a key goal of conservation genetics, which is important for efficiently conserving and utilizing genetic diversity of germplasm resources. Geographical events and climate changes play a critical role in determining the historical distribution of species and reshaping the spatio-temporal pattern of population genetic variation [2,3,4], which could be further explored by investigating the genetic differentiation and relationship between the continent and island species.

Hainan Island is a continental island in southern China. It has tropical monsoon climate characteristics due to its geographic location [5]. The complex topography and favorable hydrothermal conditions have fundamentally shaped the abundant floristic diversity, which has always been a hot topic in ecological and evolutionary biology research [6,7]. Around the Eocene, Hainan Island slowly split from the adjacent Asian mainland, eventually becoming separated from the mainland by the formation of the Qiongzhou Strait [8]. Many studies have suggested that the fluctuation of sea level due to the Quaternary climatic changes led to periodic formation and inundation of a mainland bridge, which caused Hainan Island to be connected to the mainland multiple times [9,10] and enabled exchange of fauna and flora between the mainland and islands. It is argued that some species on Hainan Island arrived via dispersal or originated via dispersal-isolation-divergence during the Miocene and the Pleistocene [6,8,11]. The geographical isolation and subsequent multiple connections between the mainland and Hainan Island had a huge impact on the biota of Hainan Island, thus profoundly affecting the genetic differentiation of populations between mainland and Hainan Island. This provides a great opportunity to explore species diversification and phylogeographical pattern between island and mainland [12].

*Castanopsis* is the third largest genus of Fagaceae, with about 120 species, that are important timber trees and the main component of subtropical evergreen broad-leaved forests and tropical monsoon rainforests [13,14,15,16]. The nuts of most *Castanopsis* species are edible and contain copious amounts of water-soluble tannin; as such, they have important industrial value. *Castanopsis chinensis* (Spreng.) Hance is widespread in Guangdong, Guangxi, Guizhou, and Yunnan province of China, and shows high morphological variation [17,18]. *Castanopsis glabrifolia* J. Q. Li & Li Chen and *Castanopsis qiongbeiensis* G. A. Fu are limited to the coastal beaches of Wenchang county in the northeast of Hainan Island. Despite similar morphological characteristics among the three species, both the leaf size and the diameter of the cupules of *C. glabrifolia* and *C. qiongbeiensis* are generally smaller than that of *C. chinensis*. *C. glabrifolia* was published first as a geographical variety of *C. chinensis*, but it was treated as an independent species later [13,19]. *C. qiongbeiensis* was published first as an independent species of Hainan Island; however, it was later believed to belong to *C. chinensis* [20,21,22]. These taxonomic controversies are mainly due to a lack of comprehensive understanding of morphological variation in *C. chinensis*. Thus, it is supposed that the relationship among *C. chinensis*, *C. glabrifolia*, and *C. qiongbeiensis* could be clarified based on investigation of their genetic differentiation.

In recent years, it has become common to use DNA variation data to study population structure and genetic differentiation. Simple sequence repeat (SSR) is a commonly used molecular marker and has been widely used to investigate genetic diversity, for example in assessing the population structure and conservation units of *Castanopsis sclerophylla* [23] and species delimitation between *Castanopsis hainanensis* and *Castanopsis wenchangensis* [24]. Thus far, the genetic differentiation and relationship among *C. chinensis*, *C. glabrifolia*, and *C. qiongbeiensis* have not been evaluated. In this study, the genetic differentiation and gene flow among *C. chinensis*, *C. glabrifolia*, and *C. qiongbeiensis* were investigated by using nuclear SSRs and sampling roughly across the species’ native range in order to obtain a comprehensive understanding of their genetic relationship and the diversification process.

## 2. Results

Seven out of twenty-two loci significantly deviated from HWE (*p* < 0.01) and were excluded from further analysis. The results of genetic diversity analysis of 15 nSSRs are summarized in Table 1. The number of alleles observed (A) changed from 3 to 20. The observed heterozygosity (H_O_) and expected heterozygosity (H_E_) ranged from 0.068 to 0.805 and 0.078 to 0.884, with mean value of 0.583 and 0.665, respectively. The within population gene diversity (H_S_) and total gene diversity (H_T_) varied from 0.080 to 0.800 and 0.079 to 0.883. The overall level of genetic differentiation among populations was moderate; the average values of F_ST_, R_ST_, and G_ST_ was 0.136, 0.209, and 0.142, respectively. At the population level, the number of alleles observed (A) and allele richness (A_R_) ranged from 2.867 to 6.667 and 2.867 to 3.550 (Table 2). The gene diversity (H) was from 0.493 to 0.647. The observed heterozygosity (H_O_) and expected heterozygosity (H_E_) varied from 0.500 to 0.644 and 0.453 to 0633. The level of genetic diversity was highest in the *C. qiongbeiensis* (A = 5.747, A_R_ = 3.401, H = 0.611, H_O_ = 0.625, H_E_ = 0.595). The level of genetic diversity in *C. chinensis* (A = 4.483, A_R_ = 3.124, H = 0.588, H_O_ = 0.546, H_E_ = 0.558) was similar to that in *C. glabrifolia* (A = 4.958, A_R_ = 3.196, H = 0.564, H_O_ = 0.567, H_E_ = 0.540).

**Figure 1:**
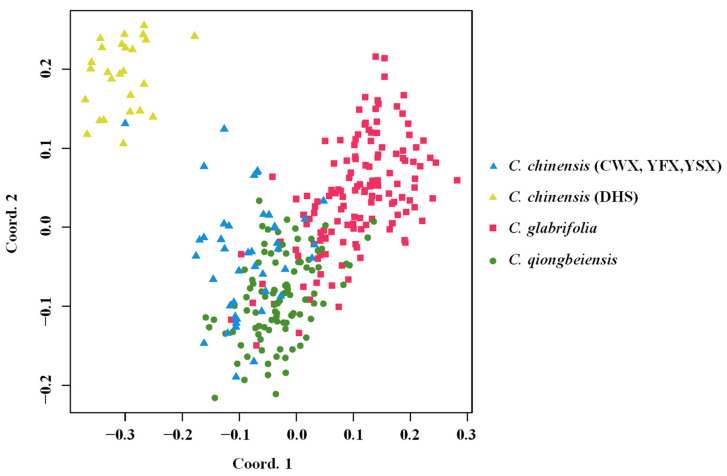
PCoA analysis for all samples of the three *Castanopsis* species based on 15 nuclear SSRs. *C. chinensis* (CWX, YFX, YSX): individuals of *C. chinensis* from populations CWX, YFX, and YSX in Guangxi Province. *C. chinensis* (DHS): individuals of *C. chinensis* from population DHS in Guangdong Province.

The result of PCoA analysis is shown in Figure 1. The first and second principal coordinates of PCoA plot accounted for 8.41% and 6.71% of the total variation, respectively. Although there were some degree of overlap, *C. chinensis*, *C. glabrifolia*, and *C. qiongbeiensis* were discriminated generally along the first coordinate axis, and *C. glabrifolia* separated from *C. qiongbeiensis* along the second coordinate axis. In addition, population DHS of *C. chinensis* differentiated from the other three populations along the second coordinate axis.

**Figure 2:**
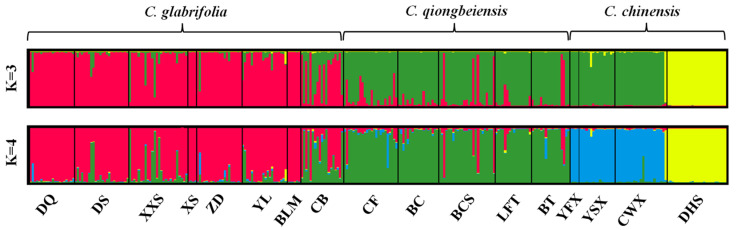
Bayesian clustering plot for all samples of the three *Castanopsis* species when K = 3 and K = 4. Each vertical bar represents a single individual, and different colors represent the population structure. Population codes are the same as in Table 4.

The optimal K value obtained in the genetic structure analysis was 4. All populations of *C. glabrifolia* and *C. qiongbeiensis* made up one gene pool, respectively (Figure 2). *C. chinensis* was divided into two clusters: population DHS alone constituted one group and the other three populations (CWX, YFX, YSX) composed another. Genetic admixture was shown in some populations, particularly in CB, indicating high level of gene flow between *C. glabrifolia* and *C. qiongbeiensis*. A suboptimal K = 3 was selected in the genetic structure analysis. In this situation, three populations of *C. chinensis* (CWX, YFX, YSX) were clustered together with *C. qiongbeiensis*. The AMOVA analysis showed that most of the genetic variation was attributed to differences within population (Table 3). There was significant genetic differentiation among *C. glabrifolia*, *C. qiongbeiensis*, and *C. chinensis* (F_CT_ = 0.076).

**Figure 3:**
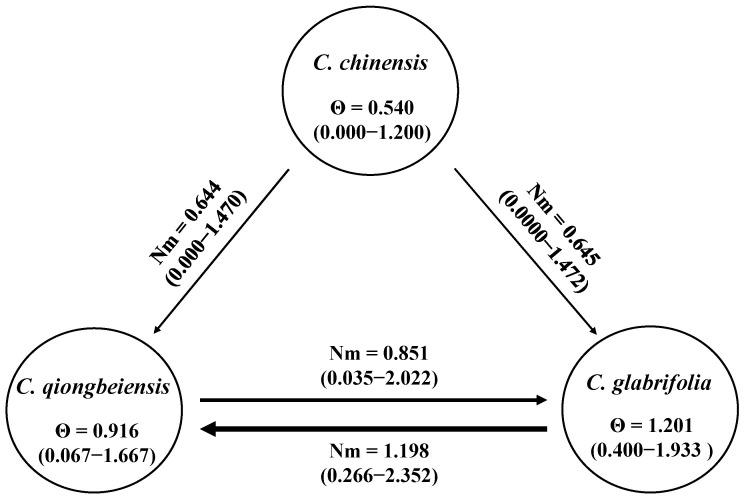
The best demographic model obtained in the present study. Θ: Effective population size; Nm: Historical gene flow; The arrow indicated the direction of gene flow, the thickness of the arrow indicated the magnitude of gene flow, and the values in parentheses indicates 95% confidence interval.

Migration model 2 obtained the highest support. In this scenario (Figure 3), appreciable unidirectional gene flow was detected from *C. chinensis* to *C. glabrifolia* (Nm = 0.6449) and from *C. chinensis* to *C. qiongbeiensis* (Nm = 0.6443), which suggested plant dispersal from the mainland to Hainan Island. Asymmetric gene flow happened between *C. glabrifolia* and *C. qiongbeiensis*. The level of gene flow from *C. glabrifolia* to *C. qiongbeiensis* was 1.1983, while the gene flow in the opposite direction was 0.8507. The effective population size was large in *C. glabrifolia* (Θ = 1.2010) and *C. qiongbeiensis* (Θ = 0.9159), but small in *C. chinensis* (Θ = 0.5404).

## 3. Discussion

The novelty of the present work is to evaluate genetic diversity and genetic differentiation among three *Castanopsis* species on Hainan Island and mainland China based on a same set of SSR markers. Genetic diversity has a significant impact on the survival and adaptation potential of species. The genetic diversity of plant populations on islands is usually lower than that on continents [25]. Limited gene flow, natural selection, and possible historical bottleneck effects may lead to a lower levels of genetic diversity in island populations [26,27]. However, our study revealed that *C. glabrifolia* on Hainan Island possessed higher genetic diversity than *C. chinensis* in mainland China (Table 2). This is mainly because Hainan Island is a continental island and *C. glabrifolia* might have been on Hainan Island before separating from the mainland. The result is consistent with other studies, where island origin and age have significant effects on the genetic diversity of island plant species [26,28]. Some plant species even originated in Hainan Island and then expanded their range to mainland China, such as *Camellia drupifera* [29]. Of course, the genetic diversity of *C. chinensis* revealed in this study may be influenced by the limited number of molecular markers and sampling. In other studies, this species has been revealed to harbor rich genetic diversity [30,31]. Our results also revealed frequent gene exchange between *C. glabrifolia* and *C. qiongbeiensis*, which might increase and maintain their genetic diversity. High level of the genetic diversity of *C. glabrifolia* would facilitate it to adapt the special island environment.

Geographical isolation and dispersal are important factors affecting plant genetic diversity and population structure. Inferring the relative importance of geographic isolation and gene flow in population differentiation can help understand the evolutionary history of island biodiversity [32]. The discontinuous distribution of plants caused by geographical isolation would restrict gene exchange among populations, increase genetic differentiation among populations, and lead to local adaptation [28,33,34,35]. The Qiongzhou Strait is a natural geographical barrier for plant populations between Hainan Island and mainland China. The role of strait isolation in population and species differentiation has been shown in previous studies [9,36]. Historical changes in sea level caused by climate fluctuation have repeatedly led to the connection of Hainan Island to mainland China [37,38], which provided opportunities for gene exchange between *C. chinensis* and *C. glabrifolia* as well as between *C. chinensis* and *C. qiongbeiensis*, since these three species have close genetic relationships.

The best demographic model showed unidirectional gene flow from *C. chinensis* to *C. glabrifolia* as well as from *C. chinensis* to *C. qiongbeiensis* (Figure 3), suggesting pollen- or seed-mediated dispersal from mainland to island. Long distance pollen-mediated gene flow is common in plants [39]. The flowering time of *C. chinensis* is from May to July; however, pollens from *C. chinensis* seem unlikely to have spread from mainland China to Hainan Island due to prevailing southeast monsoon during this period [14]. Therefore, long distance seed dispersal seems more likely to have contributed to the gene flow from mainland China to Hainan Island, just like the spread of oak acorns [40]. In contrast, the gene flow between *C. glabrifolia* and *C. qiongbeiensis* is bidirectional and more frequent, mainly because they occur sympatrically on Hainan Island. These results highlighted the isolation effect of the Qiongzhou Strait, which has played an important role in promoting genetic differentiation among the three *Castanopsis* species between Hainan Island and mainland China. Similar results were found in a recent study, which suggests that species of the *Persea* group (Lauraceae) on Hainan Island originated via a dispersal-isolation-divergence pattern [8].

Researchers have held different views about the taxonomy and genetic relationship of *C. chinensis*, *C. glabrifolia*, and *C. qiongbeiensis*. *C. glabrifolia* was originally published as a variety of *C. chinensis* (*C. chinensis* var. *hainanica*) [13]. However, Chen [19] believed that there were significant morphological differences between *C. chinensis* var. *hainanica* and *C. chinensis*, thus upgrading it from a variety (*C. chinensis* var. *hainanica*) to an independent species (*C. glabrifolia*). In this study, genetic structure analysis clearly showed that *C. glabrifolia* and *C. chinensis* were independent gene pools, thus supporting Chen’s view that *C. glabrifolia* is an independent species. *C. qiongbeiensis* was originally published as an independent species [41], but it was later considered as the same species of *C. chinensis* [22]. In this study, genetic structure analysis showed that *C. qiongbeiensis* was also an independent gene pool. However, it shared a gene pool with the Guangxi populations (CWX, YFX and YSX) of *C. chinensis* when ancestral group number (K) was defined as 3 (Figure 2), which suggested that *C. qiongbeiensis* had closer genetic relationship to *C. chinensis*, thus should be regarded as an incipient species and independent conservation unit.

*C. chinensis*, *C. glabrifolia*, and *C. qiongbeiensis* may have initially lived on a continuous landmass and had only minor differentiation. During the Pleistocene, tectonic events and climate changes made the Qiongzhou Strait a geographic barrier between mainland China and Hainan Island [8]. The isolation effect of the Qiongzhou Strait increased the genetic differentiation of species on both sides of the strait. Our results showed that there was significant genetic differentiation among *C. chinensis*, *C. glabrifolia*, and *C. qiongbeiensis* (Table 3, F_CT_ = 0.076, *p* < 0.000). However, according to Wright’s criteria [42], the differentiation index indicated a moderate degree of differentiation when it was between 0.05 and 0.15. The differentiation between *C. chinensis* in mainland China and *C. glabrifolia* and *C. qiongbeiensis* on Hainan Island may be diminished by gene flow that occurred during the historical period when Hainan Island was connected to mainland China. The repeated emergence of the land bridge between mainland China and Hainan Island provided opportunities for the plant to disperse from mainland China to Hainan Island (Nm = 0.6443–0.6449). The result was consistent with the study of *Quercus pacifica* [41] and *Eriogonum arborescens* [43] in California Channel Islands, where historical gene flow attenuated the population differentiation caused by strait isolation. On the contrary, the genetic differentiation would be high if the historical gene flow was very low (Nm = 0.000–0.004), for example, *Amentotaxus argotaenia* on Taiwan island [44] and *Nigella* species in eastern Aegean [45].

## 4. Materials and Methods

**Figure 4:**
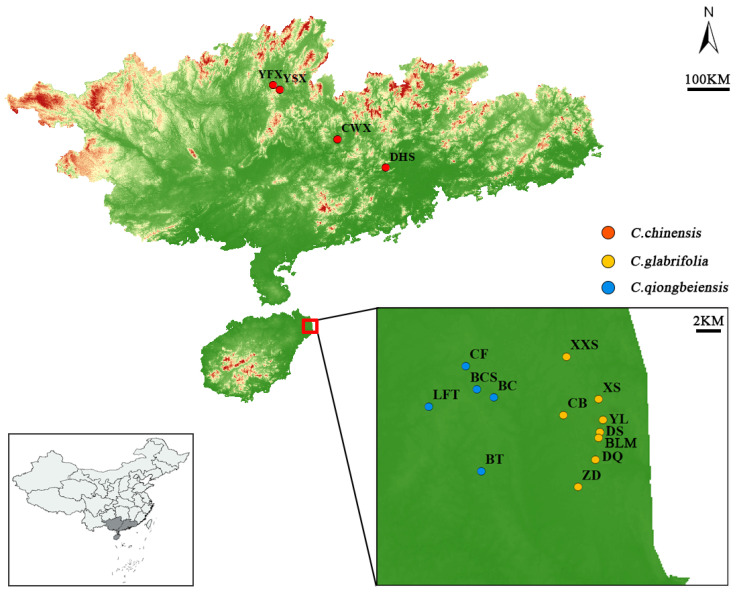
Populations of the three *Castanopsis* species sampled in the present study. The population code was the same as in Table 4.

We collected 69 samples from 4 natural populations of *C. chinensis* in Guangdong and Guangxi Provinces, 239 samples from 8 populations of *C. glabrifolia*, and 5 populations of *C. qiongbeiensis* in Hainan Island (Figure 4, Table 4). Fresh leaves were collected in the field and immediately dried with silica gel. The sampled trees were kept at least 20 m apart to avoid collecting closely related individuals due to seed reproduction.

Genomic DNA was extracted from the silica gel-dried leaves using the Tiangen Plant Genomic DNA Extraction Kit (DP320) according to the instructions of the manufacturer. A total of 15 primer pairs of nuclear SSRs (Table 5) were screened from those originally reported in *Castanopsis* and *Castanea* species [46,47,48]. Quadruple fluorescent polymerase chain reaction (PCR) was amplified using the Type-it microsatellite PCR kit (QIAGEN, Hilden, Germany). PCR was performed in a mixture including 20 ng of genomic DNA, 1× PCR Master Mix, 1× Q -Solution, and 10 μM of each primer. The forward primer was labeled with different fluorescent dyes (TAMRA, HEX, 6-FAM, and ROX). The PCR program was set as follows: 95 °C for 5 min, followed by 28 cycles of 95 °C for 30 s, 57 °C for 90 s, and 72 °C for 30 s, and a final extension at 60 °C for 30 min. The PCR products were separated by capillary electrophoresis with the ABI-3730XL fluorescence sequencer (Applied Biosystems, Foster City, CA, USA), using LIZ500 as the internal standard. Alleles were scored using Genemarker2.2.0 [49].

Deviation from Hardy–Weinberg equilibrium (HWE) was tested with 1000 permutations using FSTAT v 2.9.3 [50]. SSRs that deviated from HWE were excluded from further analysis. FSTAT v 2.9.3 [50] and GeneALEx v 6.5 [51] were used to analyze genetic diversity parameters including number of alleles observed (A), allele richness (A_R_), gene diversity (H), expected heterozygosity (H_E_), observed heterozygosity (H_O_), within population gene diversity (H_S_), total gene diversity (H_T_), genetic differentiation among populations under an infinite allele model (F_ST_), proportion of the total genetic diversity attributable to population differentiation (G_ST_), genetic differentiation among populations under a stepwise mutation model (R_ST_), and inbreeding coefficient (F_IS_).

R package “polysat” [52] was used to conduct Principal Coordinates Analysis (PCoA). Structure v 2.3.4 [53] was used to perform population genetic structure analysis. The K value was set from 1 to 17, and each was independently repeated 20 times. The length of the burn-in period was set to 1,000,000, and MCMC replications after the burn-in were set to 1,000,000. The optimal K value was selected according to the Evanno method using Structure Harvester [54]. The coefficient for cluster membership of each individual was averaged across the 20 independent runs using Clumpp v 1.1.2 [55], and the results were graphically displayed using Distruct v 1.1 [56]. Analysis of molecular variance (AMOVA) was performed using Arlequin v 3.5 [57] to determine the proportion of genetic variation partitioned within populations, among populations, and among species.

**Figure 5:**
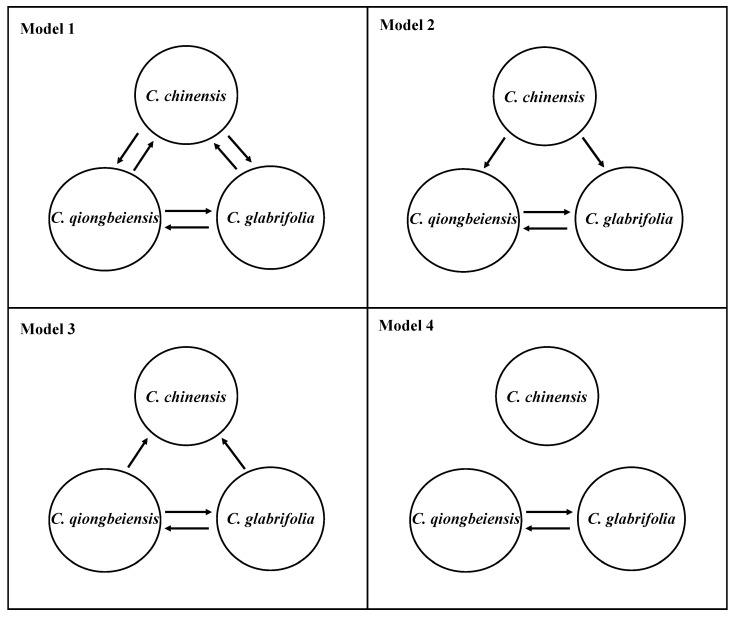
The four migration models tested in the present study. Model 2 got the highest support. Arrows represent the direction of gene flow.

Migrate-n v 4.4 [58] was used to estimate the effective population size (Θ) and migration rate (M). Formula Nm = Θ × M/x was used to calculate gene flow (Nm), where x is the fixed coefficient (x = 1 for mitochondrial genes, x = 4 for nuclear genes). Four migration models were defined by considering the possible gene flow among species (Figure 5), including (1) bidirectional gene flow among three species, (2) unidirectional gene flow from the mainland to Hainan Island and bidirectional gene flow within Hainan Island, (3) unidirectional gene flow from Hainan Island to the mainland and bidirectional gene flow within Hainan Island, and (4) bidirectional gene flow within Hainan Island but no gene flow between the mainland and Hainan Island. Migrate-n was implemented with the Bayesian inference strategy. Three independent runs were performed for each model using four parallel chains with static heating (temperature 1.0 1.5 3.0 1,000,000.0). The number of recorded steps in chain and burn-in was set to 10,000, and 100,000, respectively. The best model was selected by comparing the marginal likelihoods using thermodynamic integration in Migrate-n [59].

## 5. Conclusions

In this study, we examined the genetic differentiation and relationships among *C. chinensis*, *C. qiongbeiensis*, and *C. glabrifolia*. *C. qiongbeiensis* and *C. glabrifolia* were endemic on Hainan Island and had similar morphological characteristics to *C. chinensis* in mainland China. We revealed moderate but significant genetic differentiation among *C. chinensis*, *C. glabrifolia*, and *C. qiongbeiensis*; genetic differentiation between *C. chinensis and C. glabrifolia* was larger than that between *C. chinensis* and *C. qiongbeiensis*. Our results supported the argument that *C. glabrifolia* should be considered an independent species and argued that *C. qiongbeiensis* should be regarded as an incipient species and independent conservation unit.

## Figures and Tables

**Table 1:** Characteristics of the 15 nuclear SSRs in all samples of the three *Castanopsis* species.

Locus	A	H_O_	H_E_	H_S_	H_T_	F_ST_	F_IS_	R_ST_	G_ST_
CC-75	9	0.571	0.669	0.608	0.673	0.119	0.040	0.214	0.102
CS20	20	0.747	0.875	0.800	0.872	0.081	0.077	0.476	0.087
CS24	6	0.354	0.445	0.365	0.436	0.165	0.060	0.224	0.171
CC39198	6	0.068	0.197	0.090	0.168	0.553	0.253	0.830	0.479
CC34976	19	0.789	0.884	0.792	0.883	0.106	0.011	0.394	0.109
CC30089	7	0.591	0.705	0.516	0.703	0.253	−0.102	0.178	0.277
CC39174	15	0.630	0.750	0.664	0.759	0.129	0.045	0.130	0.131
CC3722	13	0.718	0.786	0.701	0.787	0.124	−0.031	0.159	0.114
Cch15	8	0.500	0.560	0.526	0.589	0.076	0.041	0.052	0.113
CC41684	3	0.081	0.078	0.080	0.079	0.005	−0.038	−0.009	−0.003
Ccu62F15	10	0.682	0.785	0.685	0.792	0.101	0.042	0.204	0.143
CC4950	17	0.779	0.858	0.777	0.857	0.074	0.027	0.029	0.100
CcC02022	16	0.805	0.872	0.783	0.875	0.091	−0.008	0.103	0.111
CC4562	6	0.661	0.749	0.682	0.749	0.099	0.028	0.048	0.095
CC13029	10	0.769	0.754	0.693	0.760	0.069	−0.089	0.099	0.094
mean	11	0.583	0.665	0.584	0.665	0.136	0.024	0.209	0.142

A: alleles observed; H_O_: observed heterozygosity; H_E_: expected heterozygosity; H_S_: gene diversity within populations; H_T_: gene diversity in the total population; F_ST_: genetic differentiation among populations; F_IS_: inbreeding index; G_ST_: the proportion of total genetic diversity that occurred among the population; R_ST_: genetic differentiation among populations under a stepwise mutation model.

**Table 2:** Genetic diversity parameters in 17 populations of the three *Castanopsis* species.

Species	Population	Sample Size	A	A_R_	H	H_O_	H_E_	F_IS_
*C. chinensis*	DHS	26	5.067	3.317	0.647	0.592	0.633	0.057
	CWX	23	5.533	3.205	0.567	0.538	0.554	0.026
	YFX	4	2.867	2.867	0.556	0.517	0.481	0.017
	YSX	16	4.467	3.108	0.583	0.537	0.563	0.068
	mean		4.483	3.124	0.588	0.546	0.558	0.042
*C. glabrifolia*	DQ	20	5.000	3.070	0.536	0.526	0.522	0.025
	DS	24	5.333	3.192	0.562	0.531	0.550	0.051
	XXS	26	5.933	3.294	0.576	0.574	0.565	−0.004
	XS	4	3.000	3.000	0.586	0.633	0.519	−0.032
	ZD	20	5.333	3.082	0.524	0.507	0.511	0.034
	YL	20	5.933	3.509	0.612	0.627	0.597	−0.041
	BLM	6	3.467	2.903	0.493	0.500	0.453	−0.017
	CB	19	5.667	3.516	0.622	0.642	0.606	−0.024
	mean		4.958	3.196	0.564	0.567	0.540	−0.001
*C. qiongbeiensis*	CF	24	6.667	3.550	0.622	0.644	0.610	−0.038
	BC	18	5.600	3.503	0.625	0.611	0.607	0.006
	BCS	25	6.333	3.525	0.630	0.617	0.617	0.006
	LFT	16	4.867	3.050	0.555	0.617	0.539	−0.12
	BT	17	5.267	3.376	0.622	0.635	0.604	−0.021
	mean		5.747	3.401	0.611	0.625	0.595	−0.033

A: alleles observed; A_R_: allele richness; H: gene diversity; H_O_: observed heterozygosity; H_E_: expected heterozygosity; F_IS_: inbreeding index.

**Table 3:** The AMOVA results for all samples of the three *Castanopsis* species.

Source of Variation	Sum of Squares	Variance Components	Percentage Variation	Fixation Indices (*p* < 0.000)
among species	191.068	0.391	7.610	F_CT_: 0.076
among populations within species	225.866	0.335	6.510	F_SC_: 0.071
within populations	2641.659	4.410	85.880	F_ST_: 0.141

**Table 4:** Sampling location and size of 17 populations of the three *Castanopsis* species.

Species	Population Code	Population Location	Sample Size	Longitude (°E)	Latitude (°N)
*C. chinensis*	DHS	Dinghushan	26	112°33′8.78″	23°9′51.63″
	CWX	Cangwuxian	23	111°31′58.72″	23°46′10.14″
	YFX	Yongfuxian	4	110°9′20.4″	24°55′3.47″
	YSX	Yangshuoxian	16	110°17′37.91″	24°49′45.12″
*C. glabrifolia*	XXS	Xiaoxishan	26	110°56′30.90″	19°49′52.81″
	XS	Xinshi	4	110°57′48.24″	19°48′10.94″
	CB	Changbi	19	110°56′23.54″	19°47′32.68″
	YL	Yalang	20	110°57′58.68″	19°47′22.20″
	BLM	Baolongmei	6	110°57′50.43″	19°46′51.98″
	DS	Dashan	24	110°57′47.37″	19°46′38.02″
	DQ	Dongqun	20	110°57′40.40″	19°45′45.84″
	ZD	Zhudui	20	110°56′57.93″	19°44′39.71″
*C. qiongbeiensis*	LFT	Longfeitou	16	110°50′59.25″	19°47′53.27″
	CF	Changfa	24	110°52′28.12″	19°49′30.59″
	BCS	Baocaishan	25	110°52′55.52″	19°48′34.70″
	BC	Baicai	18	110°53′36.32″	19°48′15.19″
	BT	Bangtou	17	110°53′5.85″	19°45′18.08″

**Table 5:** The 15 primer pairs of nuclear SSRs used in this study.

Locus	Repetitive Unit	Primer Sequence (5→3′)	Fragment Length	Reference
CC-75	(GA)_6_	F: <TAMRA>AACACCAGAGCTTGAGAGCG	110–138	treegenesdb.org
		R: CCTTGACATTGTCGATGGTG		
CC-39198	(AG)_11_	F: <6-FAM>GGTTGTTGTCGTTGTCGTTG	205–215	treegenesdb.org
		R: TCTGTCTCCGTTCACCCTCT		
CC-34976	(GA)_7_	F:<ROX>GTGGTGGATTTTGGGTATGG	253–291	treegenesdb.org
		R:TCCCAAACCTTGTCACCTTC		
CC-30089	(TGT)_6_	F: <HEX>ACTTGGTTCTCCGAAGCTCA	115–133	treegenesdb.org
		R:ACCGCTACTTCTTCAGCCCT		
CC-39174	(GA)_6_	F:<6-FAM>GGAGGAGGGATCATGTGAGA	219–249	treegenesdb.org
		R:TCCCAGAAATCCAAATCCCT		
CC-3722	(AG)_10_	F:<TAMRA>AGAGATGGGTTGGGAAGGTT	130–160	treegenesdb.org
		R:GGCCTCTCTGGTTTGTGTGT		
CC-41684	(ACC)_6_	F: <ROX>ATCCTCCAAGCAATCCTCCT	288–294	treegenesdb.org
		R: TCAAGTGTGTGCGAGTGACA		
CC-4950	(GT)_5_	F:<6-FAM>GCGATACCTCCAGACATGGT	246–284	treegenesdb.org
		R:CAGCTTGAAGAAATCTGGGC		
CC-4562	(TCG)_7_	F:<TAMRA>CGTATAGGGTGGAAACGGAA	144–159	treegenesdb.org
		R:GGACAAGCAAATCACGGAAT		
CC-13029	(TC)_8_	F:<HEX>CACACCTCGTTGTTTGTGCT	125–143	treegenesdb.org
		R:CGAGGAGAAGATAGGAAAAGC		
CS20	(AG)_13_	F:<ROX>AATTTCACATCCCAACTCTGCGA	246–290	[46]
		R:TGGAGGGAGTAGTGGACGATCAA		
CS24	(CAA)_6_	F:<TAMRA>ATCACCGGAGAAAACCCTAACGA	118–133	[46]
		R:AATGTTTCGGACCAATTCGAGGT		
Cch15	(CT)_11_	F:<6-FAM>CCCATAACGTCTGACCCCTA	229–251	[47]
		R:CCAAAAGGGCTTCATAACCA		
Ccu62F15	(TC)_17_	F:<TAMRA>TTGCATCCTCAGCTTTCTCA	132–156	[47]
		R: GCCCTCTCCTAACACCAATAATAC		
CcC02022	(TC)_12_	F:<ROX>TTCACTTGTTTTTCCCGACCAGA	342–372	[48]
		R:CCGCTAAAATGGTGTTGCAGAAG		

## Data Availability

The data presented in this study are available as Supplementary Materials.

## Supplementary Materials

The following supporting information can be downloaded at: https://www.mdpi.com/article/10.3390/plants13111486/s1.
